# Prevalence of a type-I interferon immune response against malaria liver stage infection

**DOI:** 10.1186/1475-2875-9-S2-P19

**Published:** 2010-10-20

**Authors:** Peter Liehl, Miguel Prudêncio, Céline Carret, Maria M Mota

**Affiliations:** 1 Unidade de Malaria, Instituto de Medicina Molecular, Faculdade de Medicina da Universidade de Lisboa, 1649-028 Lisboa, Portugal; 2 Unidade de Parasitologia Molecular, Instituto de Medicina Molecular, Faculdade de Medicina da Universidade de Lisboa, 1649-028 Lisboa, Portugal

## 

*Plasmodium* sporozoites, transmitted to the mammalian host through a mosquito bite, travel to the liver, where they invade hepatocytes and develop into a form that is then able to infect red blood cells [1]. In spite of the importance of innate immunity in controlling microbial infections, very little is known about its role during the liver stage of malaria infection.

To determine which type of innate immune response is triggered by *Plasmodium berghei* ANKA sporozoites during the liver stage of malaria we investigated transcriptome variations in this organ 40 hours after infection. We identified 69 genes, mainly classical type-I interferon stimulated genes, whose expression was induced at least 2-fold in comparison to non-infected control mice. Strikingly, the expression of all induced genes was reduced in *Plasmodium*-infected mice lacking a functional type-I interferon receptor (IFNAR1). Furthermore, we observed a 2-3 fold increase of liver parasite load in IFNAR1-deficient mice compared to their wild-type counterparts. Conversely, infection of wild-type mice after pre-stimulation of the type-I interferon response led to a reduction in liver infection.

Altogether, our data demonstrate for the first time that *Plasmodium berghei* sporozoites are able to induce a type-I interferon immune response in the liver and that this innate immune response contributes to the host resistance during the malaria liver stage. Future work will address how this immune response is activated during a liver infection by *Plasmodium* and how it inhibits the infection.
